# Cx43-Associated Secretome and Interactome Reveal Synergistic Mechanisms for Glioma Migration and MMP3 Activation

**DOI:** 10.3389/fnins.2019.00143

**Published:** 2019-03-19

**Authors:** Qurratulain Aftab, Marc Mesnil, Emmanuel Ojefua, Alisha Poole, Jenna Noordenbos, Pierre-Olivier Strale, Chris Sitko, Caitlin Le, Nikolay Stoynov, Leonard J. Foster, Wun-Chey Sin, Christian C. Naus, Vincent C. Chen

**Affiliations:** ^1^Department of Cellular and Physiological Sciences, Life Sciences Institute, University of British Columbia, Vancouver, BC, Canada; ^2^Signalisation et Transports Ioniques Membranaires (STIM), CNRS ERL 7003, University of Poitiers, Poitiers, France; ^3^Department of Chemistry, Brandon University, Brandon, MB, Canada; ^4^Department of Biochemistry and Molecular Biology, Centre for High-Throughput Biology, University of British Columbia, Vancouver, BC, Canada

**Keywords:** secretome, MMP3, osteopontin, ARK1B10, glioma, migration, connexin43, gap junction

## Abstract

Extracellular matrix (ECM) remodeling, degradation and glioma cell motility are critical aspects of glioblastoma multiforme (GBM). Despite being a rich source of potential biomarkers and targets for therapeutic advance, the dynamic changes occurring within the extracellular environment that are specific to GBM motility have yet to be fully resolved. The gap junction protein connexin43 (Cx43) increases glioma migration and invasion in a variety of *in vitro* and *in vivo* models. In this study, the upregulation of Cx43 in C6 glioma cells induced morphological changes and the secretion of proteins associated with cell motility. Demonstrating the selective engagement of ECM remodeling networks, secretome analysis revealed the near-binary increase of osteopontin and matrix metalloproteinase-3 (MMP3), with gelatinase and NFF-3 assays confirming the proteolytic activities. Informatic analysis of interactome and secretome downstream of Cx43 identifies networks of glioma motility that appear to be synergistically engaged. The data presented here implicate ECM remodeling and matrikine signals downstream of Cx43/MMP3/osteopontin and ARK1B10 inhibition as possible avenues to inhibit GBM.

## Introduction

Gliomas are cancers of the central nervous system that arise from glial-like precursor or dedifferentiated glial cells (Parkin et al., 2001; Furnari et al., 2007). The most aggressive subtype, glioblastoma multiforme (GBM), is categorized as a World Health Organization grade IV astrocytoma. Despite radical treatment, patients with GBM have an extremely poor prognosis with a median survival of 12–15 months (Parkin et al., 2001; Louis et al., 2016). GBM tumors are comprised of a heterogeneous population of tumor and host cells, that are characterized by angiogenesis, proliferation and evasion of apoptosis. High-grade gliomas further demonstrate a high propensity to invade. Furthermore, hypoxic conditions within a growing mass initiates patterns of gene expression that promotes GBM tumors to seek new niches within the CNS to expand (Demuth and Berens, 2004). The close examination of actively moving glioma demonstrates a mechanism of mutual exclusivity between the pathways regulating invasion and proliferation. In this regard GBMs appear to have a switch-like ability to delay entry into the cell cycle to migrate; while highly proliferative tumors have a disproportionate number of cells that do not invade (Giese and Westphal, 1996; Giese et al., 1996; Giese, 2003). The identification of molecular signatures of cell proliferation vs. migration in glioma would be of significant interest.

As a natural barrier for migrating glioma cells, the regulation of the extracellular matrix (ECM) and the secretome has received much attention as avenues to inhibit GBM (Levental et al., 2009; Wu et al., 2010; Cox and Erler, 2011; Lu et al., 2012). To facilitate invasion, cells of GBM are known to regulate secretion and alter the ECM of host tissues (Nakada et al., 2007; Lu et al., 2012). Previous work by Yoon et al. (2014) examined the secretome of U373MG cells, correlated to hypoxic and normoxic conditions to identify stanniocalcins (STC1 and 2), and insulin-like growth factors binding proteins that correlate with glioma grade. To help discern the secretome signature of invasive GBM, Formolo et al. provided an in-depth analysis of U118, LN18, T98, and U87 cells to identify more than 1100 proteins, among which the non-proteolytic factor chitinase-3-like protein 1 (CH3L1) was found to be linked to the invasion of U87 cells (Formolo et al., 2011).

As invasion and tumor growth are critical aspects underlying glioma, there is intense interest in identifying the molecular pathways of GBM. Previous studies in glioma have shown that Cx43 suppresses proliferation (Zhu et al., 1991; Huang R.P. et al., 1998; Fu et al., 2004). However, a growing number of recent reports have implicated Cx43 with increases in migration and invasion (Kameritsch et al., 2012; Naus et al., 2016). Gap junction proteins have also been linked to the regulation of the cytoskeleton, and the migration of neural crest cells in developing mice (Huang G.Y. et al., 1998). Cx43 is postulated to modulate these pathways via calcium signal propagation (Frisch et al., 2003), protein–protein interactions (Theiss and Meller, 2002; Bates et al., 2007; Wall et al., 2007), the establishment of intercellular junctions/channels (Wei et al., 2005; Laird, 2009) and cell-to-cell adhesion (Elias et al., 2007). In this study, we chose the rat glioma cell line C6 which expresses very low levels of endogenous Cx43 and its clone C6-13 which express high levels of exogenous Cx43 (Zhu et al., 1991). Providing evidence of paracrine signals, the application of C6-13 conditioned media increased the mobility of parental C6 cells. Owing to differences in Cx43 expression and behaviors that are typical of aggressive glioma, we sought to compare the secretomes of C6/C6-13 cells by quantitative proteomics (nanoHPLC-MS/MS). The conditioned media of C6-13 cells was found to contain significant increases in the pro-migratory proteins osteopontin and matrix metalloproteinase-3 (MMP3). This level of expression was confirmed by Western blotting and high-resolution multiple reaction monitoring (HR-MRM). Viscosity and gelatinase activity assays, alongside our use of intramolecularly quenched fluorogenic MMP3 substrates (NFF-3) finds the ECM of C6-13 cells undergoes intense remodeling. The data presented here implicate ECM activities and matrikine signals downstream of Cx43/MMP3/osteopontin as possible avenues to inhibit GBM.

## Materials and Methods

### Cells and Cell Culture

The rat C6 line (American Type Culture Collection, ATCC) maintains many characteristics of GBM (Grobben et al., 2002) and naturally expresses low levels of Cx43 (Naus et al., 1991). The choice of these cells as a glioma model is motivated by their extensive use, and recognition of cancer stem cell behavior (Zheng et al., 2007), including their ability to generate GBMs in rats upon injection in the brain (Beljebbar et al., 2010). C6-13 is a C6 clone stably expressing Cx43 that demonstrates enhanced gap junctional intercellular communication, and restricted proliferation (Zhu et al., 1991). These cells have been in routine use by ourselves and others to monitor the cellular and biochemical aspects of gap junctions – including trafficking, turnover, and regulated growth (Charles et al., 1992; Naus et al., 1993; Stout et al., 2002; Fu et al., 2004; Chen et al., 2006, 2012). Glioma cells were maintained at 37°C, 95% air and 5% CO_2_ within a humidified incubator. Cells were cultivated in high glucose (4.0 g/L) Dulbecco’s Modified Eagles Medium (DMEM; Thermo Fisher Scientific) containing L-glutamine (4.0 mM), antibiotics (penicillin, 100 IU/mL; streptomycin, 100 μg/mL) with 10% fetal bovine serum (FBS; Gibco).

### Migration (Wound Healing) Assays

Cells (C6/C6-13) were seeded at 2 × 10^5^ cells in 12-well plates (Nunc, Nucleon^TM^) and incubated (37°C, 95% air/5% CO_2_) for 3 days. Cultures at ∼80% confluence were scraped with a micropipette tip (P200, 1.1 mm OD), rinsed with phosphate buffered saline (PBS), and incubated with fresh media. Cell migration was accessed in serum-free (-FBS) or complete (+FBS) DMEM. For conditioned media transfer scrape-wound experiments, cells were taken through the same conditions, but on 6-well plates (Nunc, Nucleon^TM^). From 90% confluent cultures (DMEM, 10% FBS, 10 cm plates), conditioned media were prepared by thoroughly washing cells twice with 10 mL of PBS, followed by the application of 12 mL of fresh, serum-free media (24 h, 37°C, 95% air/5% CO_2_). Conditioned medium (2 mL/well) was transferred to C6/C6-13 cells and immediately taken through scrape wound procedures. At 4, 8, and 24 h intervals, travel distances were monitored after methanol fixation (10 min) and crystal violet stain. Images were taken on an inverted microscope (Motic AE31) equipped with a digital camera and processing software (Motic Images Plus 2.0). Pictures of 3 random areas were imaged. Scrape areas (pixels) were measured using ImageJ (Schneider et al., 2012). Areas of migration (area = y ^∗^ x) were converted to migration distance in the y-direction. As the dimensions of the field of view and length of the scrape (x-direction) were known and held constant, the migration distances (y = area/x) and velocities (*v* = y/time) were readily calculated. Distance values were expressed as a mean value, ± standard error of the mean (SEM). Distances of glioma travel were compared by Student’s *t*-test, with a significant difference at ^∗∗^*p* ≤ 0.01 and ^∗∗∗^*p* ≤ 0.001. Statistical tests were performed using Microsoft Excel and presented in GraphPad Prism (San Diego, CA, United States) or DataGraph (Visual Data Tools Inc., United States).

### Immunofluorescence

C6/C6-13 cells were seeded on coverslips (12 mm glass, Thermofisher Scientific, 2 × 10^4^ cells). Confluent cultures were taken through scrape-wound procedures, rinsed with PBS, formalin fixed, blocked with BSA (2%) and permeabilized with 0.3% Triton X-100 (in 2% BSA). Cells were then exposed for 1 h to primary antibodies directed against actin (goat polyclonal, 1:200, Santa Cruz Biotechnology) or Ki-67 (rabbit polyclonal; 1:100; Santa Cruz Biotechnology). Coverslips were incubated with anti-rabbit or anti-goat secondary antibodies (1:500) linked to FITC or TRITC and washed with PBS. Coverslips were mounted with antifade medium with DAPI (Thermofisher/Life Technologies). Cells residing at scrape borders were imaged under fluorescence and DIC microscopy (Zeiss, Axioskop, Germany).

### Conditioned Media Viscosity Measurement

Viscosity of C6/C6-13 conditioned media were measured using a capillary viscometer that constructed using a 18-gauge needle and a 1 mL syringe. Viscometer flow-through was collected under gravity using a standard beaker in a biocontainment hood. The laminar flow of 1 mL of media (24 h conditioning) was measured using a standard stopwatch. Between measurements, the viscometer was washed in 70% ethanol and dried.

### Secretome Analysis

C6/C6-13 cells were seeded in two 15 cm dishes (Nunc) containing each 20 mL of complete DMEM. At 80% confluence cell cultures were rinsed twice with serum-free DMEM (10 mL) and maintained under serum-free conditions for 24 h. Possible differences in C6/C6-13 cell death due to FBS-free media (after 24 h) was determined by the trypan blue exclusion test (Strober, 2001). Here, dead/suspended cells in spent media were aspirated, collected, and combined with adherent cells that were released by trypsinization. Cells collected by centrifugation were rinsed twice with serum-free DMEM and incubated for 3 min in trypan blue stain (0.4%). After rinsing twice with fresh DMEM, cells were counted under light microscopy using a hemocytometer. No differences in cell survival between C6/C6-13 (∼98%) was observed (Supplementary Figure S1).

For secretome isolation, conditioned media was collected in 50 mL tubes, treated with protease inhibitor cocktail (1 tablet/10 mL; Roche Applied Science, Mannheim, Germany), centrifuged (5500 rpm; 10 min; 4°C) and decanted to remove insoluble debris. Proteins were precipitated with 25 mL of ethanol (4°C, 2 h) supplemented with 40 μL/mL sodium acetate (2.5 M; pH 5.0). Tubes were centrifuged (5500 rpm; 30 min; 4°C), decanted and isolated pellets rinsed with ice cold ethanol (to remove residual protease inhibitor). Pellets were suspended in 1 mL of trypsin digestion buffer (1% sodium deoxycholate and 50 mM NH_4_HCO_3_ at pH 8). Total protein quantity was determined by BCA (Pierce/Thermo Fisher Scientific, Rockford, IL, United States). Proteins (1 mg) were then reduced with DTT (1 μg/50 μg of proteins; 30 min at 37°C) and alkylated with iodoacetamide (IAA; 5 μg/50 μg of proteins, 20 min at 37°C). Protein digestions were conducted overnight (37°C) with sequencing-grade modified trypsin (1 μg/50 μg protein; Promega, Madison, WI, United States). Peptides were enriched by C18 solid-phase extraction (International Sorbent Technology Ltd., United Kingdom) and dried. The pellets were suspended in 15 μL of sodium acetate (0.5 M; pH 5.0) and peptides were isotopically modified with formaldehyde (dimethylation) at lysine and n-termini (Hsu et al., 2003; Boersema et al., 2008; Chen et al., 2012). In brief, 15 μL of “heavy” (CD_2_O) and “light” (CH_2_O) formaldehyde (200 mM) were used to label secretome peptides from C6-13 and C6 cells, respectively. Sodium cyanoborohydride (1.0 M) was added (1.5 μL/sample, 40 min in the dark; 20°C), with a second round of labeling performed at pH to 7.5 using NaOH (aqueous, 1.0 M). Reactions were quenched with 3.0 M NH_4_Cl (15 μL/sample; 10 min in the dark; 20°C). Samples were suspended in buffer (3% acetonitrile, 1% trifluoroacetic acid, 0.5% acetic acid) and processed with C18 stage-tips. Samples were collected in a 96-microwell plate, dried and suspended in 3.2 μL of sample buffer. Labeled peptides were separated using an Agilent 1100 nanoHPLC using an autosampler (4°C). Separations were conducted on a 150 mm × 75 μm ID column (Dr Maisch, 3 μm, ReproSil-Pur 120 C18-AC), mobile phase A – 0.5% aqueous acetic acid, mobile phase B – 80% acetonitrile in 0.5% aqueous acetic acid, gradient from 6% B to 80% B at 0.2 μL/min from 20 to 85 min). Peptides were analyzed by a high-resolution linear trapping quadrupole-orbital trapping mass spectrometer (LTQ-OrbitrapXL, Thermofisher, Germany) with a generation of 1 full scan MS (Orbitrap, *m/z* 300-2,000, *R* = 60000), followed by 5 data-dependent CID MS/MS scans in the LTQ (minimum signal 1000 counts, isolation 2.0 Da, normalized collision energy of 35.0, accepting charge states +2, +3, and +4). Active dynamic exclusion was set to 3 min after the acquisition of 3 spectra. Raw datafiles associated with this study have been uploaded to ProteomeXchange.org (accession PXD012175).

### MS Data Analysis

Raw files were processed with MaxQuant software, version 1.5.5.1 (Cox and Mann, 2008) and Mascot (Matrix Science v.2.5) against the Rat Uniprot database (UP000002494, uploaded March 25, 2017). Trypsin enzyme specificity with peptides having a minimum length of 7 amino acids were considered. A maximum of one missed cleavage was allowed. Carbamidomethyl cysteine (fixed), alongside oxidation of methionine (variable) were enabled modifications for database searches. A 1% false discovery rate with the additional constraint of having been observed by both Mascot Server (v.2.6, Matrix Science) and Andromeda (MaxQuant) search engines. Relative quantitation of heavy and light peptide ratios reported by this study are based on extracted ion chromatograms (XICs) carrying the dimethyl modification. Protein identification required a minimum of two unique peptides having been observed within at least 2 biological replicates. Raw, MzXml, and mgf files, alongside and MaxQuant Protein outputs have been made available (PXD012175^1^). Statistical significance of differentially expressed proteins were interrogated using Perseus (v.1.60.7) (Tyanova and Cox, 2018). The Student *t*-test (two-tailed, unpaired, equal variance) was used to calculate significance (*P* ≤ 0.05). The threshold for significant change in expression was set to a FPR of 5%.

### High-Resolution Multiple Reaction Monitoring (HR-MRM)

Label-free quantitation of C6/C6-13 secretome peptides were performed by HR-MRM using an Agilent 1200 nanoHPLC interfaced to a quadrupole time-of-flight mass spectrometer (QTOF, Agilent 6530). Peptides were subjected to fragmentation by collision induced dissociation (CID): osteopontin (VAEFGSSEEK, collision energy = 24.64 V), MMP3 (ESVDSAIER, 15.3 V) fibronectin (FTNIGPDTMR, CE 26.05 V). Peptides were separated using a flow rate of 300 nL/min on a 20 cm × 100 μm ID column packed with C18 (Dr Maisch, 3 μm, ReproSil-Pur) using a linear gradient of 5–35% B (99.9% acetonitrile, 0.1% formic acid) over a span of 78 min (HPLC buffer A: 99.9% water, 0.1% formic acid). Peptides were quantified using Skyline (v.4.1.0) (Pino et al., 2017). Full-scan HR-MRM spectra were verified by spectral matching (NIST Rat spectral libraries)^2^ and amino acid database search (<1%FDR, Mascot v2.6, Matrix Science). Data files (.MzML, .mgf, Skyline files) associated with this analysis have been submitted to proteomexchange.org (PXD012175).

### SDS-PAGE and Western Blot Analysis

Western blot analyses were carried out on conditioned media of C6 or C6-13 cells (80% confluency) in 15 cm dishes and incubated for 24 h in serum-free DMEM. The concentration of secretome and cellular lysates proteins were determined by BCA (Pierce/Thermo Fisher Scientific, Rockford, IL, United States). Proteins from C6/C6-13 whole cell lysates or conditioned media (-FBS) were separated on 4–12% gradient mini-gels (150 V, 2 h). Gel separated proteins were transferred onto PVDF (Bio-Rad; 35 V, 1 h) and blocked (1 h) in a 5% (w/v) solution of non-fat milk powder in TBST (20 mM Tris-HCl, pH 7.4; 150 mM NaCl and 0.2% Tween-20). Membranes were incubated for 4 h with diluted rabbit polyclonal anti-MMP3 antibody (1:1000, 17873-1-AP, ProteinTech Group), or goat polyclonal anti-osteopontin (1:1000; Affinity BioReagents) antibodies in 5.0 mL of TBST containing 1% milk. Membranes were washed three times with TBST (10 min) and incubated with horseradish peroxidase-conjugated donkey anti-rabbit or anti-goat IgG (1:3000) in 1% milk TBST for 1 h, washed (TBST, 3 × 10 min). Blots were processed using standard film (ECL, GE Healthcare) or a C-DiGit Blot Scanner (ECL, Li-Cor). Thus, to ensure the validity of our loading procedures, we quantified total protein using a second SDS-PAGE gel. These Coomassie-stained gels demonstrated equivalent loads (Supplementary Figure S1) and transfer efficiency was monitored by staining membranes with ponceau S (data not shown). Transfer efficiency was measured using Ponceau S stain. Signals from ECL and Ponceau S (not shown) were quantified using Li-Cor Bioscience Image Studio Software (v.5.2).

### MMP Zymography

Protocols used for zymography were adapted from Mendes et al. (2005). In brief, 2.5 × 10^5^ cells were cultured in 6-well plates grown to 80% confluence. Conditioned media (24 h, 650 μL) proteins were concentrated using a 30 K MWCO (Pall Microsep) centrifugal spin filter and quantified by BCA. Concentrates were normalized (100 μg/lane) and diluted using SDS-PAGE loading buffer lacking DTT, without boiling. Electrophoresis was conducted at 4°C in 10% SDS-PAGE gels supplemented with 0.1% gelatin (Thermo Fisher Scientific). Post-separation, gels were rinsed in 18 Mohm water, followed by zymography wash buffer (incubation buffer containing 2.5% Triton-X100, 2 × 40 min) with rocking (5 min). In a sealed vessel, gels were completely submerged in incubation buffer (50 mM Tris-HCl, pH = 7.5, 5 mM CaCl_2_, 1 μM ZnCl_2_, 0.02% Brij-35, and 0.02% NaN_3_) for 24 h at 37°C. Gels were exposed to Coomassie G250 (0.5%, 1 h), distained in 18 Mohm water and 2% acetic acid fixative and imaged. Gelatinolytic activity was observed as a clear band against a blue background. Images were captured using a digital camera (α6000, Sony) and a standard light box. Zymography experiments were performed in triplicate.

### Fluorogenic Assay for MMP3 Activity

Cells were plated at a concentration of 1.0 × 10^5^ per well (6-well plates) and grown for 24 h in 2 mL of DMEM containing 10% FBS. DMEM (Sigma, D2902, 2 mL) lacking FBS was then added and allowed to condition the media for 24 h. Conditioned media was then collected and distributed into a 96-well assay plate preloaded with 15 μL of a stock solution containing NFF-3 (100 μM in DMSO). Conditioned or control media (300 μL) was added to each well (final concentration NFF-3 = 4.76 μM, MW = 1675.8 g/mole) using a standard 96 well plate. MMP3 activity was monitored at 37°C using a multi-well plate fluorimeter (SpectraMax M2, Molecular Devices). The proteolytic activity of C6/C6-13 conditioned media was measured relative to the background fluorescence produced by unconditioned DMEM. Wavelengths for excitation (325 nm) and fluorescence emission (393 nm) were measured every 10 min over 6 h.

### Bioinformatics and Analysis of Functional Networks

Functional classification and cellular outcomes of expression were evaluated through bioinformatics using Ingenuity Pathways Analysis (IPA, Qiagen) (Kramer et al., 2014). Datasets re-examined here include our previous Cx43 protein–protein interactions originating from C6-13 (Chen et al., 2012). This dataset is available via BioGrid^3^ (Stark et al., 2006) and the original publication (Chen et al., 2012) and proteomeXchange.org (PXD012175). Synergistic functions common to Cx43 interactome and secretome were determined using statistically enriched cellular pathways in IPA. Here, statistical significance of C6/C6-13 datasets (secretome and Cx43 protein–protein interactions) was determined by comparison to networks obtained from similar-sized pool of random proteins. Databases used for this analysis integrated the BIND, DIP, MIPS, BIOGRID, INTACT, and COGNIA, alongside the curated IPA Knowledgebase (Kramer et al., 2014). In addition to network enrichments, we utilized Causal Analysis feature in IPA (a.k.a. Z-score Activation) to explain/predict the direction and impact Cx43 interactors and secretome protein expression have on increasing/decreasing cell migration and predicted cellular functions. While still depending on statistics, the IPA Causal Analysis approach leverages knowledge about genetic function and rather than solely relying on network association (Kramer et al., 2014). Data from our integrated analysis of the C6/C6-13 secretome and protein–protein interactions of Cx43 were presented using Microsoft Excel. Proteins found within the secretomes of C6/C6-13 were also evaluated using the Human Cancer Secretome Database (Feizi et al., 2015) and SignalP (Petersen et al., 2011; Nielsen, 2017).

## Results

### Cx43 Expression Increases Migration of C6 Glioma Cells

**FIGURE 1:**
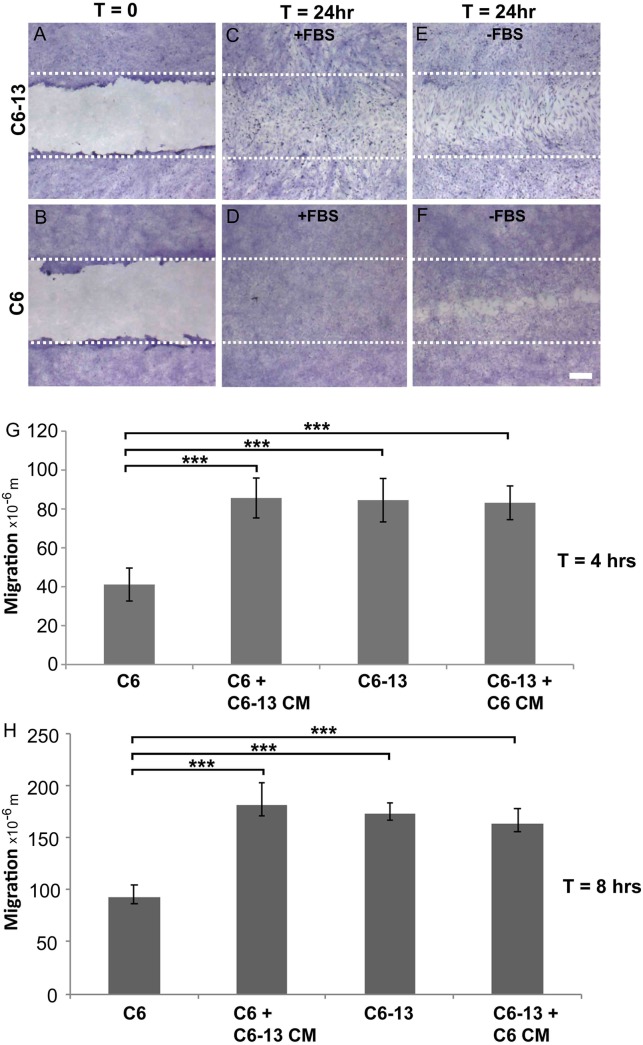
**(A–D)** Connexin expression increases migratory phenotype. **(A,B)** Confluent cultures of C6 and C6-13 cells underwent procedures for scrape-wound with representative images captured at *T* = 0 h. **(C,D)** At *T* = 24 h, in the presence of FBS, both C6 and C6-13 cells resulted in complete closures of the wound. Differences in cell density and morphology are noted. C6-13 cells are elongated, even within the unscraped regions that indicated their movement into the newly created space. Under the same conditions, this greatly contrasted C6 cells that appeared small and compact. **(E,F)** To differentiate effects of migration from cell proliferation, C6 and C6-13 cells were monitored in the absence of growth factors contained within FBS. Full recovery was only observed for C6-13, but not for C6. The white lines represent the limits of the original scrape. Cells were fixed with methanol and stained with crystal violet. Bar: 250 μm. **(G,H**) Cell migration monitored *T* = 4 and *T* = 8 h in the absence of FBS ± conditioned media (CM) from alternate cell type. Migration of C6 cells was less than C6-13 cells at both 4 and 8 h. C6 exposed to C6-13 conditioned media increase migration to levels similar to C6-13. C6-13 cell were not impacted by C6 conditioned media. Results are shown from three different sets of experiments, encompassing 6 scrapes and 18 measurements. Distance values were expressed as a mean value, with error bars representing the (±) standard error of the mean. Significance levels: ^∗∗∗^*p* ≤ 0.001.

The expression of Cx43 is widely regarded as a potent inhibitor of glioma growth (Zhu et al., 1991; Naus et al., 1992; Sin et al., 2012). The C6 clone 13 line (C6-13) demonstrates a dramatic reduction of cell proliferation, in both *in vitro* (Zhu et al., 1991) and *in vivo* models of GBM (Naus et al., 1992). Glioma motility was monitored using the scrape wound healing assay (Figure 1A–F). At 24 h serum (Figure 1C) and serum-free (Figure 1E) conditions, scrape wound gaps were fully closed by C6-13, whereas C6 closure was only obtained after growth stimulation with FBS (Figure 1D,F). Despite having restricted cell proliferation (Zhu et al., 1991; Naus et al., 1992), wounds for C6-13 were consistently closed, an effect that was independent of FBS (Figure 1E compared to Figure 1F). In these images C6-13 are also distinguishable – polarized and elongated in the direction of migration, whereas C6 cells appeared smaller and compact. Due to the complete closure of C6-13 at 24 h, wound closures at 4 and 8 h were measured to determine the absolute distance and velocity (Figure 1G,H). This analysis confirmed the motility of C6-13 (21 μm/h) is significantly higher than C6 control (11 μm/h).

### Conditioned Media of C6-13 Contains Pro-Migratory Paracrine Signals

To determine the influence of extracellular factors produced by high motility gliomas, we next carried out conditioned media transfer experiments. While the conditioned media from C6 cells demonstrated little-to-no impact, the secretome of C6-13 cells was found to increase the motility of the parental line (Figure 1G,H). These findings suggest C6-13 factor that promote glioma migration.

### Morphological Assessment Reveals Changes in the Actin Cytoskeleton

**FIGURE 2:**
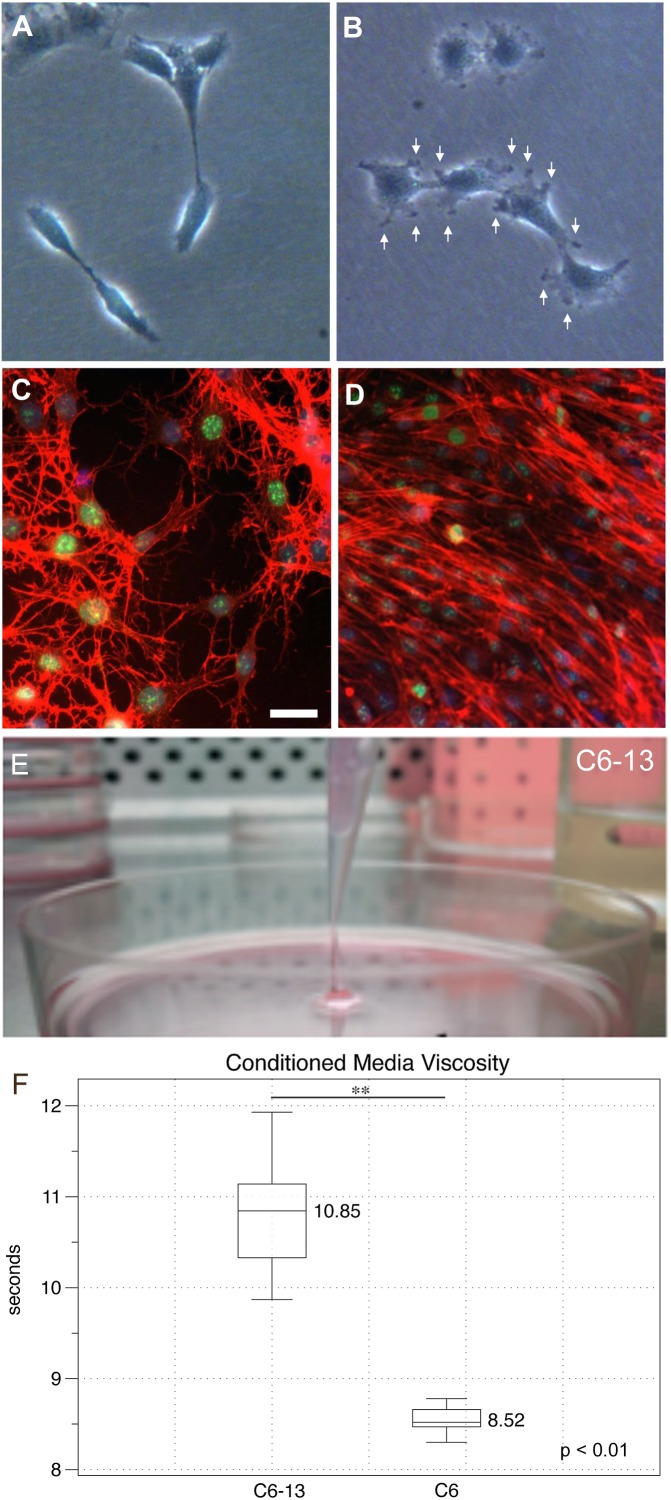
Morphology, actin organization and conditioned media of C6/C6-13. Subconfluent cultures of C6 **(A)** and C6-13 **(B)** cells. C6 present a spindle appearance, while C6-13 are flat and exhibit membrane ruffles and protrusions (arrows). **(C,D)** Actin (red) organization under scrape-wound conditions (-FBS, 15 h). **(C)** C6 actin stress fibres presented a stellate, random orientation at the wound gap. **(D)** C6-13 actin fibres were aligned between cells and parallel with the movement into the space. Nuclei were co-stained by DAPI (blue) and the cell proliferation marker Ki-67 (green). Bar: 20 μm. **(E)** C6-13 conditioned medium exhibited a higher viscosity that was readily observed during aspirations. **(F)** Conditioned media viscosity measurements. Box and whisker plot of timed laminar flow demonstrate increased viscosity of C6-13 conditioned media. Whiskers designate absolute max/min values (*N* = 8); significance using Student’s T (^∗∗^*p* ≤ 0.01).

Cancer cells rely on their actin cytoskeleton to orchestrate movements through the ECM via the generation of protrusions in their plasma membranes (Machesky, 2008). Under subconfluent conditions, cells were examined by light microscopy. At low densities, C6 cells were elongated presenting fewer cellular contacts (Figure 2A). C6-13 cells on the other hand were larger and flat, with numerous plasma membrane ruffles that resembled lamellipodia and/or filopodia-like structures (Figure 2B). To examine the actin cytoskeleton during migration, cells were subjected to wound-healing prior to fixation and actin staining. Here, the stellate cytoskeletons of C6 cells (Figure 2C) greatly contrasted with the extended appearance and directional alignment of C6-13 (Figure 2D). Morphological features observed with C6-13 are consistent with aggressive glioma cells.

### Secretome Changes Associated With C6 Glioma Motility

During exchanges of spent media, we noted that C6-13 produced a viscous medium that resembled a loose gel. We were able to aspirate this gel-like media at distances >2 cm from the surface of the culture plate (Figure 2E). Timed viscosity measurement demonstrated significant increases in the mechanical resistance of C6-13 conditioned media (Figure 2F).

**FIGURE 3:**
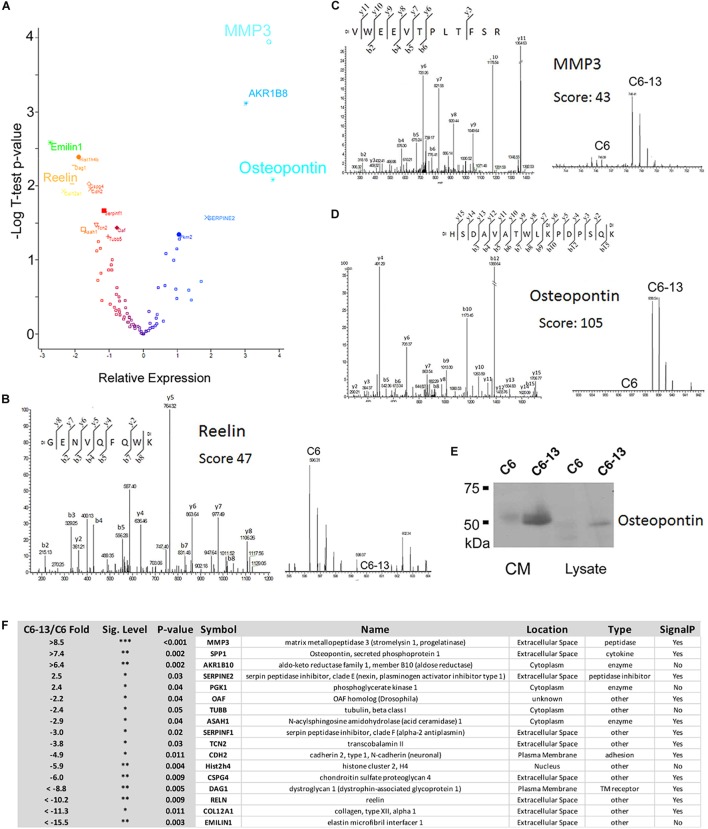
Secretome analysis identifies differentially expressed proteins. **(A)** Volcano plot of secretome proteins (*T*-test *P*-value, and log_2_ ratio) to identify differential expression. These proteins include reelin, MMP3 and osteopontin. Select MS/MS and MS spectra: **(B)** Reelin; **(C)** MMP3; and **(D)** Osteopontin showing identity and differential increases with C6/C6-13. **(E)** SDS-PAGE/Western blot of lysates and conditioned media (CM) supports increases in osteopontin in C6-13. **(F)** Table summarizing proteins found to be differentially expressed: fold-change ratio, *P*-value, anticipated cellular location, function/enzyme type and presence of a protein secretion signal.

Based on these observations, we next sought to investigate the proteins differentially secreted by C6/C6-13. In four biological replicates, peptides were covalently modified by “light” or “heavy” dimethyl (Chen et al., 2012). The full list of proteins identified are provided (Supplementary Table S1). Significance (*p* ≤ 0.05) was accessed by Student’s T using Perseus (Tyanova and Cox, 2018). Volcano plot and table of differentially regulated proteins are shown in Figure 3A,F. This analysis revealed significant increases in 5 proteins: matrix metalloproteinase-3 (MMP3, >+8.5-fold), osteopontin (SPP1, >+7.4-fold), aldo-keto reductase family 1, member B10 (ARK1B10 >+6.4-fold), serpin family E member 2 (Serpine2, +2.5-fold) and phosphoglycerate kinase 1 (PGK1, +2.4-fold); and significant decreases in 12 proteins: elastin microfibril interface-located protein 1 (EMILIN1, < -15.5-fold), collagen type XII alpha 1 chain (COL12A1, < -11.3-fold), reelin (RELN, < -10.2-fold), dystroglycan 1 (DAG1, < -8.8-fold), chondroitin sulfate proteoglycan 4 (CSPG4, -6.0-fold), histone cluster 2 H4 family member A (Hist2h4, -5.9-fold), cadherin 2 (CDH2, -4.9-fold), transcobalamin-2 (TCN2, -3.8-fold), serpin family F member 1 (SERPINF1, -3.0-fold), N-acylsphingosine amidohydrolase 1 (ASAH1, -2.9-fold), tubulin beta chain (TUBB, -2.4-fold), and out at first homolog (OAF, -2.2-fold). Representative MS and MS/MS spectra are provided: reelin (RELN, GENVQFQWK, Ratio < -10, Figure 3B), MMP3 (VWEEVTPLTFSR, Ratio > +10, Figure 3C), and osteopontin (HSDAVATWLKPDPSQK, Ratio > +10, Figure 3D). SDS-PAGE/Western blot was employed to confirm expression. MMP3 and osteopontin were quantified as “heavy-only” peptides suggesting their sole expression with C6-13. Peptides form fibronectin from this dataset demonstrated a ratio of ∼1:1 suggesting their equivalent expression between C6 and C6-13 cells. Relative measurement of osteopontin by Western blot (Figure 3E) confirmed increases this protein in C6-13 whole cell lysates and conditioned media. A summary of secretome proteins significantly changed with Cx43 expression is provided in Figure 3F.

### Targeted Detection of Osteopontin and MMP3 by HR-MRM

**FIGURE 4:**
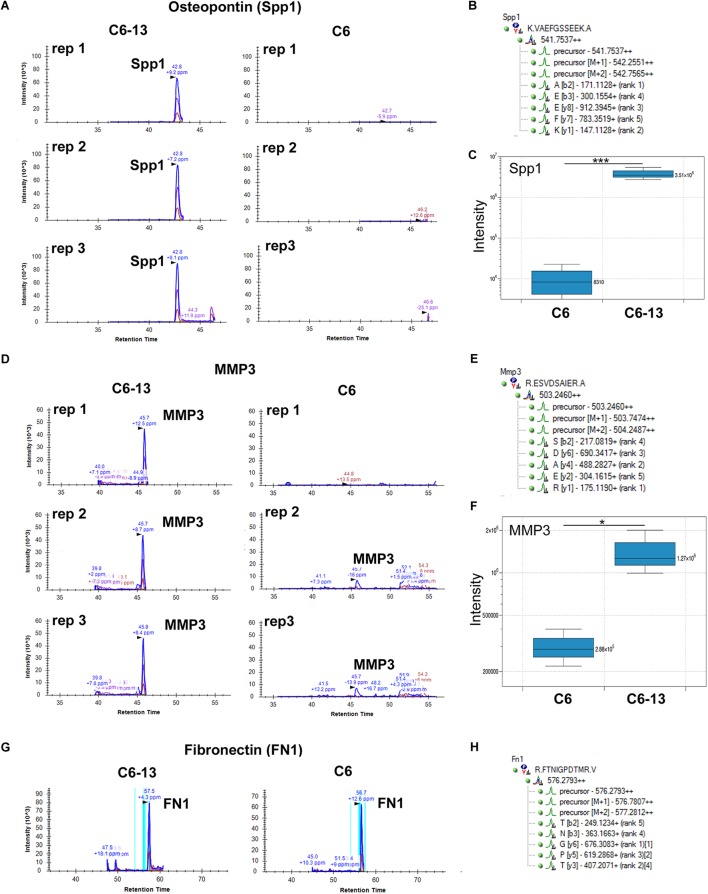
Binary increases of osteopontin and MMP3 in high motility C6-13. Targeted detection of **(A)** osteopontin/SPP1 and **(D)** MMP3 monitored by HR-MRM in biological triplicates of C6/C6-13 conditioned media. **(B,E)** Sequence, parent and fragment ions (*m/z*) used to monitor MMP3/osteopontin proteins. Significant increases of **(C)** osteopontin and **(F)** MMP3 are observed with C6-13 secretome (^∗^*p* ≤ 0.05, ^∗∗∗^*p* ≤ 0.001). **(G,H)** Fibronectin observed in C6/C6-13 was used to monitor sample load and signal normalization.

Motivated by these findings we sought to validate the expression of osteopontin and MMP3 by HR-MRM (Shi et al., 2016). HR-MRM detections were based on LC retention time, parent ion mass-to-charge *(m/z*), CID collision energy (Q2) and full-scan high resolution MS/MS (TOF) spectra. Chromatographic traces from 3 biological replicates (Figure 4) demonstrate osteopontin and MMP3 in the media of C6-13, and the near-absence of these proteins in the media of C6 cells (Figure 4C,F). Chromatographic traces, sequences, and *m/z* (parent and selected daughter b/y-ions) are shown (Figure 4B,E,H). Peptides from fibronectin were used to normalize total signal area from these chromatographic profiles (Figure 4G).

### MMP3-Mediated Proteolytic Activity in Motile Glioma

**FIGURE 5:**
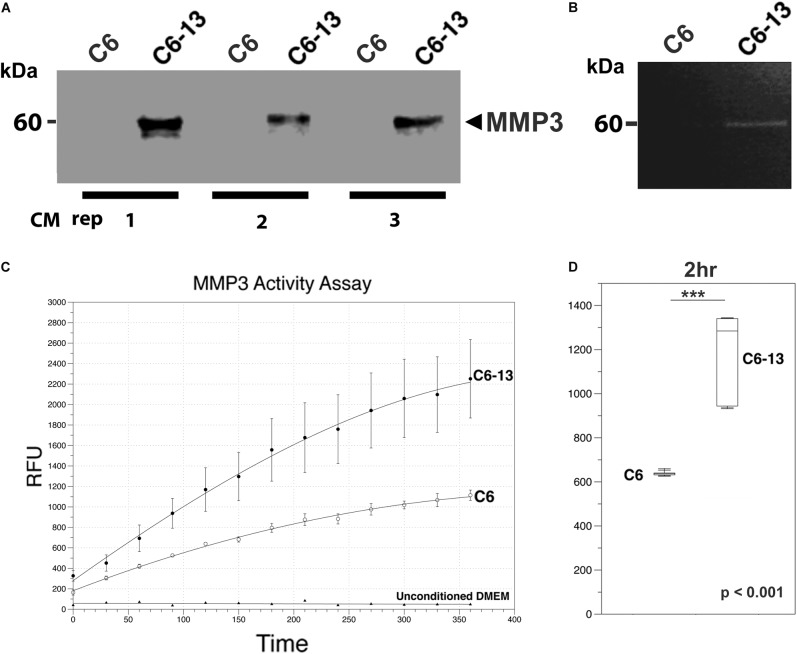
MMP3 is active in the ECM of high motility glioma. **(A)** SDS-PAGE/Western blot demonstrates the binary increases of MMP3 in C6-13 vs. C6 glioma conditioned media (CM) in 3 biological replicates. **(B)** Increases in gelatinase activity associated with MMP3 is observed in the conditioned media of C6-13, but not C6 cells. **(C)** Cleavage of the MMP3-specific fluorgenic substrate (NFF-3) demonstrates increased proteolytic activity in C6-13 CM. Limited cleavage of NFF-3 is observed with C6. Fresh, unconditioned media served as a negative control for each time point. **(D)** Representative, quantitative values from the 2 h time point demonstrates robust increases in MMP3 activity with C6-13.

MMP3 was robustly observed in the conditioned media of C6-13, but lacking with C6 cells (Figure 4D,F). To confirm these findings, we next utilized a polyclonal rabbit antibody (ProteinTech Group, 17873-1-AP) raised against the full-length sequence of MMP3 (minus the secretion signal peptide, A.A. 1-25). Consistent with our datum thus far, MMP3 was observed in the conditioned media of C6-13, but not C6 cells (Figure 5A). To ensure equal loading, BCA quantified protein were accessed using parallel run SDS-PAGE gels stained with Coomassie (Supplementary Figure S1). Using discovery and targeted proteomics (HR-MRM, QTOF), and SDS-PAGE/Western blot analysis, these findings demonstrate osteopontin and MMP3 are selectively expressed with C6-13.

Based on these findings, we next asked if the expression of MMP3 translated into proteinase activities within the conditioned medium of C6-13. To address this, we conducted a zymographic analysis and confirmed the gelatinolytic activity corresponding to the mass of MMP3 detected by Western blotting (representative figure shown, Figure 5B). To provide additional lines of evidence, we also monitored the protease using the fluorogenic substrate NFF-3 (excitation/emission = 325/393 nm) (Nagase et al., 1994). NFF-3 is a highly specific substrate having high selectively for MMP3 (kcat/Km = 218,000 s^-1^ M^-1^), and to a much lesser degree, MMP9 (kcat/Km = 10,000 s^-1^ M^-1^) (Nagase et al., 1994). Over the course of 6 h, the conditioned medium of C6-13 demonstrated significant, robust activity (Figure 5C,D). As some NFF-3 signal was observed with C6, suggests the presence of other MMPs (Zhang et al., 2003; Sahin et al., 2005). This analysis demonstrates increased rates of C6-13 migration coincides with dramatic increases in MMP3 and proteolytic activity within the ECM.

### Biological Function, Localization, and Cellular Pathways

**FIGURE 6:**
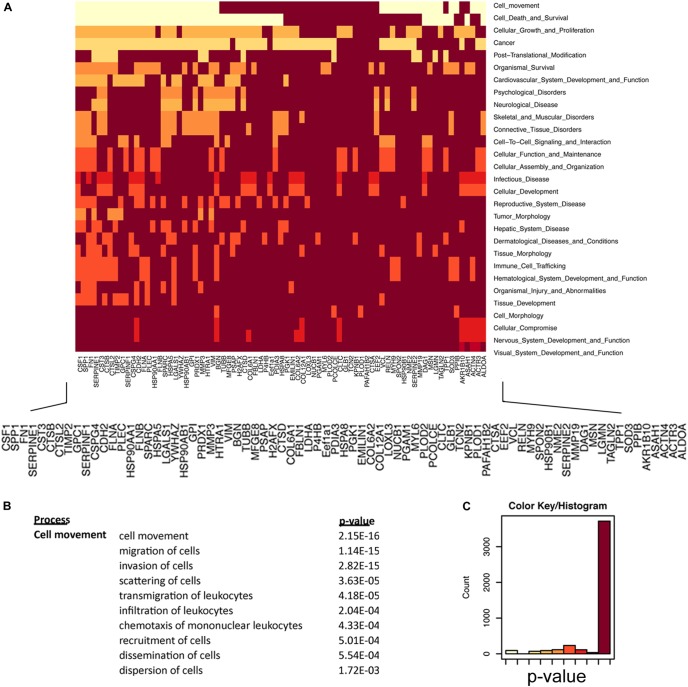
Regulators of cellular movement are significantly enriched in glioma secretome. **(A)** Heat map representation of secretome proteins and functional relationships. Lighter colors in this heat map group proteins that are statistically enriched for the listed cell function (y-axis). Protein network enrichment and statistical significance were determined by Ingenuity Pathways^TM^. **(B)** Cell Movement (*P* = 2.15 × 10^-16^) was the most statistically significant network encoded by the secretome of C6/C6-13 cells. Migration subnetworks (and *P*-values) comprising the Cellular Movement category. **(C)** Color bin histogram representing the distribution of both significant (x-axis: left, light) and non-significant (right, dark) protein networks represented in Figure 6A.

How the ECM is dynamically engaged in invasive glioma is poorly understood. Our experiments demonstrate that C6-13 cells increase viscosity and secrete promigratory factors that appear to engage pathways for glioma migration. We used an integrated proteomic and informatic strategy to examine the functional pathways and networks that are downstream of Cx43. Of the 91 total proteins identified by our secretome study (full list provided, Supplementary Table S1), 84 were annotated within the IPA Knowledge Base and considered further. SignalP (version 4.1, stringency 0.6)^4^ (Petersen et al., 2011; Nielsen, 2017) observed 45 of these proteins having signals consistent with their production and export via the protein secretion pathway (Supplementary Table S1). Further inspection of our data using the Human Cancer Secretome Database (HCSD)^5^ (Feizi et al., 2015) finds 98.8% (83/84) of C6/C6-13 proteins have been identified within the secretome of other cancers (Feizi et al., 2015); with 88% (74/84) observed with other GBM studies (Kashyap et al., 2010; Formolo et al., 2011; Gupta et al., 2013; Supplementary Table S1). Secretomes of both cell lines had strong functional links to Cellular Movement (*P* = 2.15 × 10^-16^), Cell Death and Survival (2.13 × 10^-15^), Cancer (1.87 × 10^-12^), and PTM (post-translational modification) (2.46 × 10^-11^) (Figure 6A,C). Examination for the Cellular Movement subcategory identifies proteins linked to the Migration, Invasion, and Scattering associated with the secretome of C6/C6-13 (Figure 6B).

Previous studies in glioma have demonstrated Cx43 has roles in the suppression of tumor growth (Zhu et al., 1991; Huang R.P. et al., 1998; Fu et al., 2004) and increases invasion (Kameritsch et al., 2012; Naus et al., 2016). Despite this growing body of evidence implicating connexins with GBM malignancy, the mechanism of how Cx43 impacts glioma is poorly understood. We have found C6-13 demonstrates behaviors typical of aggressive glioma – increased migration, changes in the actin cytoskeleton, increased expression of osteopontin and MMP3, and ECM remodeling via increased of MMP activity. The 17 differentially expressed secretome proteins were evaluated to determine links to phenotypic changes that were observed with Cx43. As outlined in Table 1, the top 6 functional annotations and disease processes differentiated by the Cx43 secretome (by rank) Metastasis (*P* = 5.51 × 10^-7^, Activation Z-Score +0.132), Aggregation of Cancer Cells (*P* = 3.36 × 10^-6^), Disruption of Cytoskeleton (*P* = 4.00 × 10^-6^), Metastasis of Tumor cell lines (*P* = 4.99 × 10^-6^, Activation Z-Score = +0.132), Breast/Colorectal Cancer (*P* = 5.57–5.97 × 10^-6^) and Advanced Malignant Tumor (*P* = 9.13 × 10^-6^, Activation Z-Score = +0.297).

**Table 1:** Pathways analysis of the 17 differentially regulated secretome proteins found in C6-13. Where possible, the activation *Z*-score functions of the ingenuity pathways tool was used to predict the biological impact of C6-13 expression.

Categories	Diseases or Functions Annotation	*p*-Value	Predicted Impact	Activation *z*-score	Molecules	#
Cancer, organismal injury and abnormalities	Metastasis of cells	5.51E-07	Increased metastasis	0.132	ASAH1, CDH2, MMP3, RELN, SERPINF1, SPP1	6
Cell-to-cell signaling and interaction	Aggregation of cancer cells	3.36E-06			CDH2, SERPINF1	2
Cellular assembly and organization, cellular compromise	Disruption of cytoskeleton	4.00E-06			CDH2, CSPG4, DAG1	3
Cancer, organismal injury and abnormalities	Metastasis of tumor cell lines	4.99E-06	Increased metastasis	0.132	ASAH1, CDH2, MMP3, SERPINF1, SPP1	5
Cancer, organismal injury and abnormalities	Breast or colorectal cancer	5.57E-06			AKR1B10, ASAH1, CDH2, COL12A1, CSPG4, DAG, EMILIN1, MMP3, OAF, PGKl, RELN, SERPINE2, SERPINFl, SPPl, TCN2, TUBB	16
Cancer, organismal injury and abnormalities	Breast cancer	5.97E-06			CDH2, COL12A1, CSPG4, EMILIN1, MMP3, OAF, PGK1, RELN, SERPINF1, SPP1, TUBB	11
Cancer, organismal injury and abnormalities	Advanced malignant tumor	9.13E-06	Tumor progression	0.297	ASAH1, CDH2, MMP3, RELN, SERPINE2, SERPINF1, SPP1, TUBB	8
Cellular development	Differentiation of nervous system	1.08E-05			ASAH1, CDH2, DAG1, RELN, SERPINE2, SERPINF1	6
Protein synthesis	Metabolism of protein	1.35E-05	Reduced metabolism	-1.165	CDH2, CSPG4, DAG1, HIST1H4B, MMP3, RELN, SERPINE2, SPP1	8
Cell death and survival	Apoptosis of endothelial cell lines	1.40E-05			CDH2, SERPINF1, SPP1	3
Cell-to-cell signaling and interaction	Aggregation of cells	1.41E-05	Reduction of cell aggregation	-1.959	CDH2, MMP3, RELN, SERPINE2, SERPINF1	5
Organismal functions, organismal injury and abnormalities	Closure of wound	1.47E-05			CDH2, EMILIN1, SPP1	3
Cellular movement, connective tissue development and function	Migration of cardiac fibroblasts	2.01E-05			SERPINF1, SPP1	2
Tissue development	Formation of basal lamina	2.01E-05			CSPG4, DAG1	2
Cellular development, nervous system development and function	Differentiation of neuroglia	2.35E-05			CDH2, DAG1, RELN, SERPINE2	4
Cellular movement	Scattering of cells	3.02E-05			CSPG4, RELN, SPPl	3
Skeletal and muscular system development and function	Metabolism by bone	3.07E-05			MMP3, SPP1	2
Cell-to-cell signaling and interaction	Anchoring of cells	3.68E-05			DAG1, SPP1	2
Cancer, organismal injury and abnormalities	Spindle cell carcinoma	4.35E-05			CDH2, MMP3	2
Cell-to-cell signaling and interaction	Binding of tumor cell lines	4.72E-05	Reduction of Tumor Cell Binding	-0.181	CDH2, DAG1, EMILINl, SERPINF1, SPP1	5
Tissue development	Organization of extracellular matrix	4.77E-05			DAG1, EMILIN1, MMP3, SPP1	4
Cell-to-cell signaling and interaction	Detachment of cells	5.06E-05			RELN, SERPINE2, SPP1	3
Cancer, organismal injury and abnormalities	Secondary tumor	5.17E-05	Promotion of secondary tumor	0.297	ASAH1, CDH2, MMP3, RELN, SERPINF1, SPP1, TUBB	7
Cardiovascular system development and function	Angiogenesis	5.31E-05	promotion of angiogensis	0.567	CDH2, CSPG4, EMILIN1, MMP3, PGK1, SERPINF1, SPP1	7
Organismal injury and abnormalities	Wound	5.79E-05			CDH2, EMILIN1, MMP3, SPP1	4
Cellular movement	Dispersion of cells	5.85E-05			RELN, SPP1	2
Cellular movement	Invasion of cells	5.93E-05	Increased invasion	0.176	CDH2, CSPG4, MMP3, RELN, SERPINE2, SERPINF1, SPP1	7
Nervous system development and function	Morphology of nervous system	5.96E-05			CDH2, CSPG4, DAG1, MMP3, RELN, SERPINE2, SPP1	7
Cancer, gastrointestinal disease, organismal injury and abnormalities	Colorectal cancer	6.01E-05			AKRB10, ASAH1, CDH2, COL12A1, CSPG4, DAG1, EMILIN1, MMP3, PGK1, RELN, SERPINE2, SPP1, TCN2, TUBB	14
Cellular assembly and organization, cellular function and maintenance	Formation of microtubules	8.70E-05			RELN, SERPINF1, TUBB	3
Cardiovascular disease, developmental disorder, neurological disease	Intracranial arteriovenous malformation	1.17E-04			CDH2, MMP3	2
Cell-to-cell signaling and interaction, tissue development	Binding of extracellular matrix	1.31E-04			EMILIN1, SERPINE2, SPP1	3
Cancer, endocrine system disorders, organismal injury and abnormalities	Follicular papillary thyroid carcinoma	1.53E-04			SERPINF1, SPP1	2
Cellular movement	Invasion of tumor cell lines	1.55E-04	Increased invasion	0.025	CDH2, CSPG4, MMP3, SERPINE2, SERPINF1, SPP1	6
Cancer, organismal injury and abnormalities, respiratory disease	Non-small cell lung carcinoma	1.86E-04			AKR1B10, ASAH1, COL12A1, CSPG4, MMP3, RELN, SPP1, TUBB	8
Cellular assembly and organization, cellular compromise	Disruption of actin cytoskeleton	1.95E-04			CDH2, CSPG4	2
Cancer, organismal injury and abnormalities	Benign Tumors	2.08E-04			CDH2, C0L12A1, MMP3, RELN, SERPINE2, SPP1	6
Cancer, organismal injury and abnormalities	Mesothelioma	2.08E-04			CDH2, CSPG4, SPP1	3
Cell morphology, cell-to-cell signaling and interaction	Morphology of intercellular junctions	2.08E-04			CDH2, DAG1, MMP3	3
Cancer, organismal injury and abnormalities	Hormone-refractory malignant neoplasm of pr	2.09E-04			MMP3, SPP1	2
Cancer, organismal injury and abnormalities	Breast or pancreatic cancer	2.22E-04			CDH2, C0L12A1, EMILIN1, MMP3, OAF, PGK1, RELN, SERPINE2, SERPINF1, SPP1, TUBB	13
Cancer, organismal injury and abnormalities	Poorly differentiated adenocarcinoma	2.41E-04			CDH2, SPP1	2
Cancer, gastrointestinali disease, organismal injury and abnormalities	Progression of digestive organ tumor	2.41E-04			CDH2, SPP1	2
Cancer, organismal injury and abnormalities, tumor morphology	Progression of tumor	2.43E-04			CDH2, SERPINF1, SPP1, TUBB	4
Cell-to-cell signaling and interaction	Adhesion of tumor cell lines	2.59E-04	Reduced adhesion of tumor cells	-0.625	CDH2, DAG1, EMIUN1, SPP1	4
Cell death and survival	Necrosis	2.87E-04	Increased necrosis	0.213	AKR1B10, ASAH1, CDH2, CSPG4, DAG1, MMP3, SERPINE2, SERPINF1, SPP1, TUBB	10
Cellular assembly and organization	Organization of plasma membrane	2.98E-04			CDH2, DAG1, TUBB	3
Cellular movement	Cell movement of cancer cells	3.09E-04			CDH2, CSPG4, SPP1	3
Cellular movement	Migration of keratinocyte cancer cell lines	3.48E-04			MMP3, SERPINF1	2
Cellular development, cellular growth and proliferation	Cell proliferation of tumor cell lines	3.60E-04	Increased proliferation	0.839	AKR1B10, ASAH1, CSPG4, EM1LN1, SERPINF1, SPP1, TCN2, TUBB	8
Cancer, organismal injury and abnormalities	Metastasis of carcinoma	3.88E-04			MMP3, SPP1	2
Connective tissue development and function	Cartilage development	3.93E-04			C0L12A1, CSPG4, SPP1	3
Cellular development, cellular growth and proliferation	Proliferation of keratinocyte cancer cell lines	4.08E-04			EMILIN1, SERPINF1	2
Cellular function and maintenance	Function of osteoblasts	4.08E-04			COL12Al, SPPl	2
Cancer, organismal injury and abnormalities	Advanced extracranial solid tumor	4.19E-04			CDH2, MMP3, SERPINE2, SPPl, TUBB	5
Cell morphology. organ morphology,	Morphology of muscle cells	4.90E-04			CDH2, C0L12A1, DAG1, SPP1	4
Connective tissue development and function	Strength of bone	5.20E-04			COL12A1, SPP1	2
Cell death and survival	Apoptosis of granulosa cells	5.20E-04			CDH2, SERPINF1	2
Cell-to-cell signaling and interaction, cellular assembly and organization	Cell-cell contact	5.29E-04			CDH2, DAG1, MMP3, RELN, SERPINF1	5
Cellular movement	Cell movement	5.36E-04	Increased cell movement	0.998	CDH2, CSPG4, DAGl, MMP3, RELN, SERPINE2, SERPINFl, SPPl, TUBB	9


**FIGURE 7:**
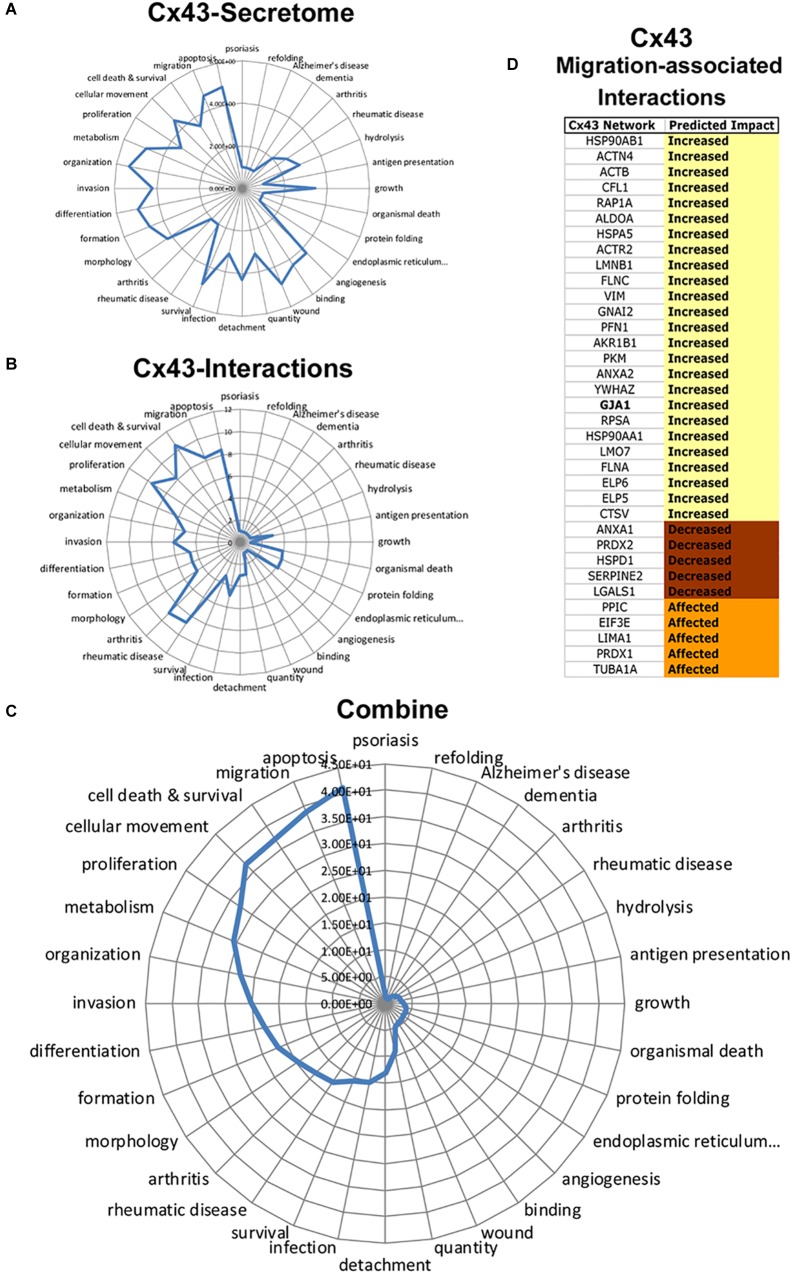
Integrated analysis of Cx43 secretome and Cx43 protein–protein interactions reveals synergistic networks. Radar plots of pathways associated with the Cx43 **(A)** secretome, and **(B)** interactome plotted as -log *P*-value. **C)** Analysis of Cx43 networks demonstrates the coherence of “Apoptosis,” “Migration,” “Cell Death and Survival,” “Cellular Movement,” and “Proliferation” between Cx43-protein interactions and secretome. **(D)** Downstream impact analysis (IPA) identifies interactions of Cx43 implicated with migration. Based on IPA informatics (Activation Z-Score) the majority of these interactions are expected to increase migration.

To determine Cx43 intracellular signals, we next conducted a retrospective analysis of immunoprecipitated Cx43–protein interactions obtained from C6-13 glioma cells (Chen et al., 2012). Figure 7 summarizes the functional annotations and disease processes for Cx43 (secretome, Figure 7A) and the connexin proteome (Cx43–protein interactions, Figure 7B). Top functions associated with Cx43 interaction in order of rank implicate networks for Cell Death and Survival (*P* = 2.99 × 10^-11^), Migration (5.15 × 10^-9^), Cellular Movement (6.79 × 10^-9^), Proliferation (3.16 × 10^-10^), and Apoptosis (3.06 × 10^-9^).

To determine synergistic impact of the Cx43 secretome and protein–protein interactions, we next utilized an approach to calculate overlapping *P*-values:

$$\text{Synergistic Score}=-\log[P-{\text{value Cell Function}}_{Cx43-\sec retome}]\times -\log\left[P-{\text{value Cell Function}}_{Cx43-\text{interaction}}\right]$$

Synergistic networks identified by this approach find cellular pathways of: Apoptosis (Synergistic Score = 41.3), Migration (38.9), Cell Death and Survival (37.3), Cellular Movement (36.9), Proliferation (32.7), and Metabolism (30.8) (Figure 7C). Downstream Effect Analysis (Kramer et al., 2014) of interactions linked to migration demonstrated an overall abundance of factors that are expected to increase migration (25/35, 71.4%, Figure 7D), with the remaining predicted to decrease (5/35, 14.3%) or generally affecting migration (5/35, 14.3%). These findings predict the intersecting pathways of Cx43 conspire to increase migration in glioma by concentrating pro-migratory proteins to the cortical surface of the cell and the degradation and remodeling of the ECM.

## Discussion

Cancer cell invasion requires the coordination of cellular programs regulating epithelial-mesenchymal transition, motility, and remodeling of the ECM. The most prominent feature underlying GBM malignancy is drug resistance and the ability of tumor cells to invade. A key goal of glioma research has been to seek a deeper understanding of the tumor microenvironment with the overall goal of developing approaches to limit infiltration (Giese and Westphal, 1996). C6 cells expressing connexin demonstrate lower cell proliferation that maintain a commonly held view of Cx43 being an inhibitor of GBM (Zhu et al., 1991). However, a number of recent reports have uncovered a “connexin paradox” in which Cx43 is a promoter of glioma malignancy (Sin et al., 2012; Naus et al., 2016). Amongst these studies, Cx43 has been found to increase migration and invasion, and resistance to the frontline chemotherapeutic agent Temozolomide (TMZ) (Gielen et al., 2013; Munoz et al., 2014; Murphy et al., 2016; Grek et al., 2018; Wang et al., 2018). Based on this body of knowledge, peptidomimetics that modulate the functions of Cx43 are currently being evaluated as adjuvants to address drug resistance in GBM (Grek et al., 2018). A better understanding of how Cx43 impacts GBM may provide avenues and novel approaches for therapeutic design.

The microenvironment influences tumor growth and behavior, including the progression and invasion of GBM. The microenvironment represents a highly dynamic niche by which glioma cells takeover the cellular programs that are normally reserved for development, tissue remodeling and repair (Quail and Joyce, 2017). As a complex meshwork, the tumor microenvironment is represented by stromal and tumor cell-derived cytokines, growth factors, enzymes and matrix proteins. Increases in glioma migration/invasion often coincide with aggressive cellular phenotypes, including alterations in cell morphology, cytoskeleton and adhesion (Lin et al., 2002). Increases in glioma migration are frequently accompanied by the secretion of regulatory matricellular proteins such as tenascin (Deryugina and Bourdon, 1996) and osteopontin/SPP1 (Jan et al., 2010). Cx43 has been shown to promote migration by wound healing (Bates et al., 2007), Transwell^TM^ assay (Bates et al., 2007) and brain slices (Oliveira et al., 2005), and regulate expression of MMP-2, MMP-9 (Zhang et al., 2003), osteopontin (Naus et al., 2000), CCN1 (Naus et al., 2000), and CCN3/NOV (Sin et al., 2008). Despite the growing body of evidence linking the gap junction protein to the control of migration and the ECM, the mechanisms of Cx43-induced migration have yet to be resolved. The findings presented here provide several lines of evidence by which Cx43 may influence GBM. In particular, this study corroborates changes in glioma motility, morphology and cytoskeletal organization with expression of Cx43 (Bates et al., 2007; Crespin et al., 2010), and is also the first study to uncover secretome and ECM mechanisms downstream of Cx43 as a driver of malignancy. Providing insight, we noted C6-13 significantly changed the viscosity and thickness of conditioned media. Indeed, the tension of cell substrates has been shown to increase migration of a number of human and rat gliomas, including the C6 line (Hegedus et al., 2006; Ulrich et al., 2009). Changes in viscosity would also foreseeably limit free diffusion of soluble factors (enzymes, chemokines, cytokines and matrikines) resulting in the formation of concentration gradients that maybe used as chemotactic and/or repulsive signals (Manini et al., 2018). Such mechanisms occurring with C6-13 may be used to guide migration and direction of glioma invasion.

To better understand the paracrine signals in glioma, we sought to compare proteins within the conditioned media of C6/C6-13 using stable-isotope labeling/high resolution LC-MS. Global proteins identified were categorized into 7 groups: peptidases, peptidase inhibitors, cytokines, adhesion, and transmembrane receptors/other (Supplementary Table S1). Secretome proteins accessed by IPA identified the enrichment of cellular processes: (1) Cell Movement, (2) Proliferation, and (3) Survival. Dissemination of the cell movement category (*P* = 2.15 × 10^-16^) revealed networks having close linkages to migration (1.14 × 10^-15^) and invasion (2.82 × 10^-15^). While the migration/invasion pathways linked to the global secretome is intriguing, it is important to note the majority of proteins identified (97/114) were considered to be unchanged between high and low motility cells (Supplementary Table S1). Indeed, the majority (88%) of the proteins characterized have been identified within other GBM secretomes studies, presumably with gliomas of differing rates of mobility. Such congruence between secretomes suggest the ECMs of high-grade gliomas are “primed” – but remain quiescent in the absence of specific cell migration signals. In light of an accumulating body of evidence linking connexins to GBM malignancy, including our observation of Cx43 conditioned media increases glioma motility, it is reasonable to suggest the Cx43 and/or ECM proteins downstream of it may serve this role. In particular, we identify 17 (5 up- and 12 down-regulated proteins) differentiating the secretome of C6-13 (Figure 3F). Indeed, Z-Score Activation implicates the Cx43 as a driver of cell migration, tumor progression and malignancy (Table 1). Of the cellular functions reporting pathway activation direction, the Cx43 secretome is predicted to increase Metastasis, Tumor Progression, Secondary Tumor Formation, Angiogenesis, and Invasion; and decreased probability of Protein Metabolism, Aggregation, and Tumor Cell Binding. These findings predict the involvement of the Cx43 secretome in migration and malignancy of GBM.

Our study shows that high motility gliomas expressing Cx43 differ from their parental counterparts by the upregulation of MMP3, osteopontin, aldo-keto reductase family 1 member B10 (ARK1B10), and to a lesser degree SERPINE2 (2.5-fold, *P* = 0.03), and phosphoglycerate kinase 1 (2.4-fold, *P* = 0.04). As migratory behavior is a hallmark of GBM, we focused on components having links to this process. In particular, there is a strong correlation between patient outcome with the activity of proteases residing within the extracellular space (Friedl and Wolf, 2008; Ren et al., 2015; Sun et al., 2015; Gong et al., 2016; Jia et al., 2017). The activity of ECM proteases such as MMPs are widely considered to be major determinants of patient outcome (Lu et al., 2012). Signals making up the MMP protease-web include the activation/inactivation of growth factors, shedding of cell surface adhesion molecules, the release of ECM-bound cytokines and growth factors, and the activation of cryptic peptides (Lopez-Otin and Overall, 2002). Cryptic peptides signals (a.k.a. matrikines and matricryptins) are generated by the precision degradation of the ECM (e.g., collagen and laminin), and matricellular proteins, such as osteopontin and SPARC (Wells et al., 2015; Eckhard et al., 2016). A perfect example of matrikine signals regulating migration/invasion is the signaling nexus mediated by MMP3/osteopontin (Agnihotri et al., 2001). Osteopontin is a secreted, sialic acid-rich glyco/phosphoprotein (Denhardt and Noda, 1998) that plays a major role in cancer progression (Chong et al., 2012), and the *in vivo* invasion of glioma cells (Jan et al., 2010). MMP3-cleaved osteopontin acquires new functions, one of which is to regulate adhesive and migratory stimuli through cell surface integrins (Agnihotri et al., 2001; Kon et al., 2014). Matricryptic signals encoded by MMP3/osteopontin have been shown to expose RGD sequences that bind integrins and thus affect adhesion, migration, and survival (Ruoslahti, 1996). Suggesting Cx43 may engage similar pathways, quantitative proteomic datasets identified the dramatic, near binary (on/off) expression of MMP3 and osteopontin. These increases were substantiated by SDS-PAGE/Western blot and targeted HR-MRM analyses. Our findings are consistent with the observation of MMP3 at invasive fronts of GBM tumors, and the reduction of invasion potential after MMP3 loss (Jin et al., 2013). Based on this data we propose matrikine signals downstream of Cx43/MMP3/osteopontin as a possible avenue to inhibit GBM.

Synthesized as inactive zymogens, cross-talk between proteases are required for the removal of the auto-inhibitory domain of MMPs, resulting in the catalytic activation and amplification of degradative signals (Sevenich and Joyce, 2014). These activities are further potentiated by interactions with other proteins, including natural inhibitors of MMP (e.g., TIMPs) (Wang et al., 2000; Lu et al., 2004). In light of the multiple layers of MMP control, we sought to survey the activities of the enzyme within the ECM materials. This study is the first to implicate ECM-remodeling and the protease-web down-stream of MMP3 with Cx43 motility. Here, gelatin zymography and loss of MMP3-specific substrates confirmed this activity within the conditioned media of C6-13 cells. In keeping with this view, it is important to note a significant number of ECM components including collagen alpha 12 (COL12A1), reelin (RELN), elastin microfibril interface 1 (EMILIN), and chondroitin sulfate proteoglycan (CSPG4) (Figure 3F) were lost and/or down-regulated with Cx43. We speculate such losses, especially within the context of high MMP3 activity, may encode molecular events underlying movement in C6 glioma cells. Experiments to investigate MMP3 substrates are currently underway.

In addition to MMP3/osteopontin, our proteomic datum identifies the up-regulation of ARK1B10 within the Cx43 secretome. The AKRs are a super family of NADPH-dependent oxidoreductases that metabolize a wide variety of carbohydrates, steroids, prostaglandins, aldehydes, ketones, and xenobiotic compounds (Matsunaga et al., 2012). AKR1B10 is a 36 KDa reductase that shares a 71% identity and α/β-barrel topology with the better studied AKR1B1 (Gallego et al., 2007). The AKR1 subfamily of enzymes have a critical role in the detoxification of cytotoxic compounds by the reduction of aromatic and aliphatic aldehydes, carbonyl compounds, and drug ketones (Matsunaga et al., 2012). Studies employing drug resistant cell lines have provided some evidence implicating members of the ARK superfamily with the acquisition of therapeutic resistance in a variety of cancers (Matsunaga et al., 2012). In medullablastoma and colon cancers, AKR1B10 has been shown to be involved with the metabolic deactivation of the chemotherapeutic agents cyclophosphamide (Bacolod et al., 2008) and mitomycin-c (Matsunaga et al., 2011). Our identification of AKR1B10 within the Cx43 secretome provides clues into possible mechanisms of GBM malignancy related to motility/invasion and TMZ resistance (Gielen et al., 2013; Munoz et al., 2014; Murphy et al., 2016; Grek et al., 2018; Wang et al., 2018). The cytotoxic effects of TMZ are largely mediated by a key carbonyl that undergoes decarboxylation and ring opening to produce the methyldiazonium ion that in turn alkylates DNA leading to cell death (Saleem et al., 2003). Although the exact role of ARK1B10 in TMZ metabolism is currently unknown, the chemical conversion of this carbonyl group to an alcohol (or further reduced form), would inhibit decarboxylation and thus methyldiazonium formation. Furthermore, these findings of ARK1B10 in the Cx43 secretome also open the possibility of gliomas utilizing Cx43 to not only increase migration but additionally set up ECM defenses to inactivate therapeutic agents prior to their arrival at gliomas. Monitoring the metabolic conversion of TMZ in the secretome and media of cells expressing Cx43/ARK1B10, alongside the testing of ARK1/ARK1B10 inhibitors (Huang et al., 2016) as possible adjuvants, would seem to be logical next steps.

Given our observed changes in the glioma secretome, plasma membrane protrusions and the reorganization of the actin cytoskeleton, we sought to characterize networks associated with Cx43. We anticipate changes occurring with Cx43 expression would be accompanied by a significant rewiring of signaling networks regulating glioma. Examinations of Cx43/gap junction channel-dead mutants, truncations, and pharmacological gap junction channel blockers have isolated the effects of Cx43-induced migration to the long c-terminal tail (Bates et al., 2007; Crespin et al., 2010) - the principle site of of Cx43 protein-protein interaction (Giepmans, 2004). Here, a large variety of regulatory factors are known to co-localize with Cx43 at gap junctions (Giepmans, 2004; Laird, 2009). We therefore examined C6-13/Cx43 co-IPs (Chen et al., 2012). Cx43 protein–protein interactions demonstrated the statistical enrichment of pathways having known functions in cell migration. Migration-associated Cx43 interactome highlights components of the actin-based cytoskeleton (alpha-actinin-4, actin (ACTB, ACTR2), filamin, and vimentin) alongside regulators of cytoskeletal dynamics (cofilin and profilin, Figure 7D). These proteins belong to a pool of accessory proteins that are mechanistically responsible for the scaffolding, polymerization and depolymerization of actin filaments, including the crawling and locomotion of cancer cells (Yamaguchi and Condeelis, 2007; Pollard and Cooper, 2009). This overlap of proteins with Cx43 suggest the gap junction protein may influence migration by the trafficking and/or scaffolding of cytoskeletal proteins to the plasma membrane or sites of intercellular adhesion. This study identifies secretome/protein–protein interaction networks that further strengthen Cx43 as a driver of motility and therapeutic resistance in glioma.

## Author Contributions

QA, MM, and VC designed the study and wrote the manuscript. NS, LF and VC conducted mass spectrometry. JN and VC conducted MS data analysis and informatics. P-OS and MM conducted microscopy and migration assays. EO, AP, CS and CL performed cell culture, zymography and NFF-3 assays. CN, LF, W-CS and VC provided reagents, feedback and conceptual advice. CN, W-CS, LF, and VC secured funding and research infrastructure. All authors discussed the results and commented on the manuscript at all stages.

## Conflict of Interest Statement

The authors declare that the research was conducted in the absence of any commercial or financial relationships that could be construed as a potential conflict of interest.

## Footnotes

1 proteomeXchange.org

2 https://chemdata.nist.gov

3 thebiogrid.org

4 http://www.cbs.dtu.dk/services/SignalP/

5 www.cancersecretome.org

## Abbreviations

Cx43: connexin43
ECM: extracellular matrix
GBM: glioblastoma multiforme
TMZ: Temozolomide
