# Supplementary figures and images for: Cx43-Associated Secretome and Interactome Reveal Synergistic Mechanisms for Glioma Migration and MMP3 Activation
Source: Front Neurosci. 2019 Mar 19;13:143. doi: 10.3389/fnins.2019.00143 (PMC6433981; doi:10.3389/fnins.2019.00143)

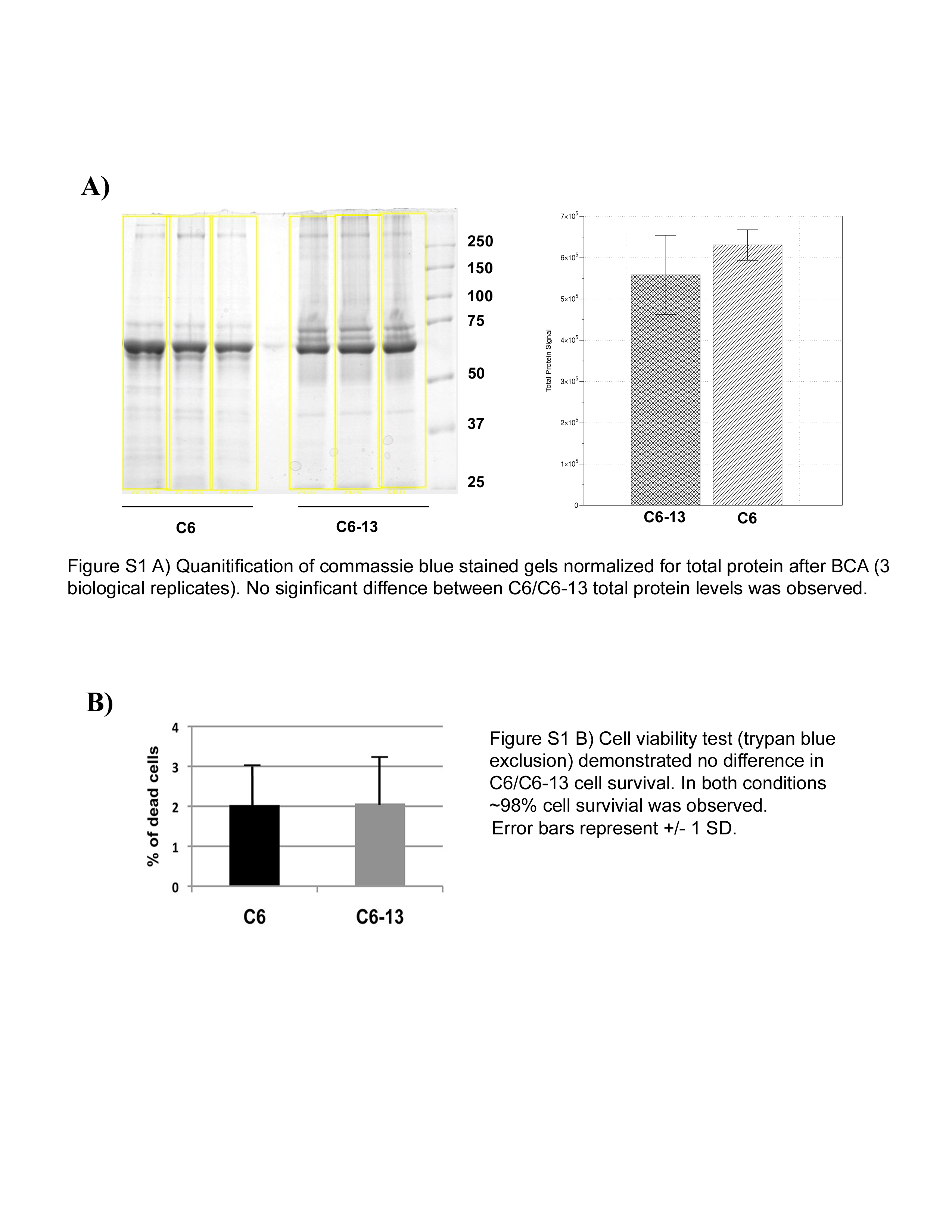

Supplement: Supplementary file 2 [file Image_1.tif]
